# Untire: an all-embracing self-management eHealth program to cope with cancer-related fatigue

**DOI:** 10.3332/ecancer.2018.ed81

**Published:** 2018-03-20

**Authors:** Abraham Johannes Kuiper, Maria Dorothea Jacoba Wolvers, Dora Vonk, Anton Hagenbeek

**Affiliations:** 1 Tired of Cancer BV, Lucasbolwerk 6, 3512 EG, Utrecht, The Netherlands; 2 Helen Dowling Institute, Professor Bronkhorstlaan 20, 3723 MB Bilthoven, The Netherlands; 3 Academic Medical Center, Department of Hematology, University of Amsterdam, Meibergdreef 9, 1105 AZ Amsterdam, The Netherlands

**Keywords:** cancer-related fatigue, psycho-education, cognitive behavioural therapy, mindfulness, physical activity, stepped care, blended care, mHealth

## Abstract

Cancer-related fatigue (CRF) is a frequent and invalidating problem in (former) cancer patients. If there are no medical causes, relief of fatigue can be attained by means of non-pharmacological interventions. Guidelines prescribe a multimodal approach with a focus on mental processes, physical activity and sleep. Online interventions have been shown to be effective in reducing CRF. These results inspired the creation of an all-embracing app on the various behavioural and physical activity modification themes that are recommended in oncological guidelines.

This basis for the ‘Untire’ app is the Daily Program, consisting of 4 components.

In 2018, the ‘Untire’ app will be launched in several languages throughout the European Union and some other countries outside Europe, such as the USA. Its effectiveness will be studied by means of a randomized controlled trial.

## The extent and impact of cancer-related fatigue

Cancer-related fatigue (CRF) is a frequently reported problem for (former) cancer patients. CRF is very common during cancer treatment [15], and is not always relieved post-treatment. CRF disturbs daily functioning and severely affects quality of life [2]. Around 3.8 million Europeans are diagnosed with cancer every year. It is estimated that 30%-40% of all (former) cancer patients suffer from prolonged and severe fatigue [17].

CRF is rarely an isolated symptom [2], it often co-occurs with pain, distress and sleep disturbance. These symptoms are probably not only co-occurring, but strongly related and can result in vicious circles. This can explain why social, occupational, and emotional functioning are often (severely) affected in (former) cancer patients who suffer from fatigue.

## Current care

CRF is underdiagnosed and undertreated [13]. Many patients think or are told that it is part of the deal and that they have to learn to live with it. But ‘how’ is the unanswered question for most of them. It seems that CRF is rarely discussed in the specialist’s office, and pushed aside when no physical, treatable cause is apparent. The National Comprehensive Cancer Networks (NCCN) published well-elaborated Standards of Care on CRF [2]. Within the differential diagnosis, research into medical factors such as anemia, cardiac failure, thyroid problems, apnea, dyspnoea, renal failure, and the like has priority. In the event that such factors can be found, a medical intervention will be started initially. If there are no indications for any of these factors, a multimodal approach is recommended that focuses on mental processes (reduction of stress, anxiety, and depression), physical activity (better energy balance) and sleep. Psycho-education, both for patients and their social environment, should be part of the interventions. Such a multimodal approach reflects that the cause of fatigue during post-treatment is unclear and likely multifactorial [2]. Multimodal non-pharmacological interventions seem to be rather effective.

Various behavioural interventions have been shown to reduce fatigue, and decrease its impact on the quality of life of patients during and after cancer [5, 7, 8, 11, 14]. Examples include cognitive behavioural therapy [4, 6], exercise training [10, 16], and mindfulness-based cognitive therapy [9]. Significant effects can be attained if these interventions are directed at those who are most severely fatigued [12], but they do not have sufficient reach to be of use to the large and growing numbers of patients who suffer from cancer-related fatigue and associated symptoms. This is because the interventions are face-to-face, labour-intensive and hard to scale up.

Online interventions can make care accessible to a wider population outside the environments of institutions. Such online interventions, with a therapist in the background, are effective in reducing fatigue [1, 3] and show potential for unguided interventions.

These findings inspired two of the authors (Kuiper and Vonk) to create an all-embracing app on the various behavioural and physical activity modification themes that have been shown to be effective in guided interventions and/or are recommended in the NCCN guidelines. In this app, called ‘Untire’, users follow their own daily path of learning and experiencing various themes and exercises, to gain tools for regaining energy and improving quality of life.

It took some time to develop the content, design, (interaction) developers, and financial means. After starting with the creators’ own savings and having overcome several setbacks, the project was finally subsidized with a Horizon 2020 SME Phase II Grant and gained real momentum after that. The moon-shot mission is to help at least 1 million fatigued (former) cancer patients worldwide within the next five years.

## The ‘Untire’ app

**Figure 1.**
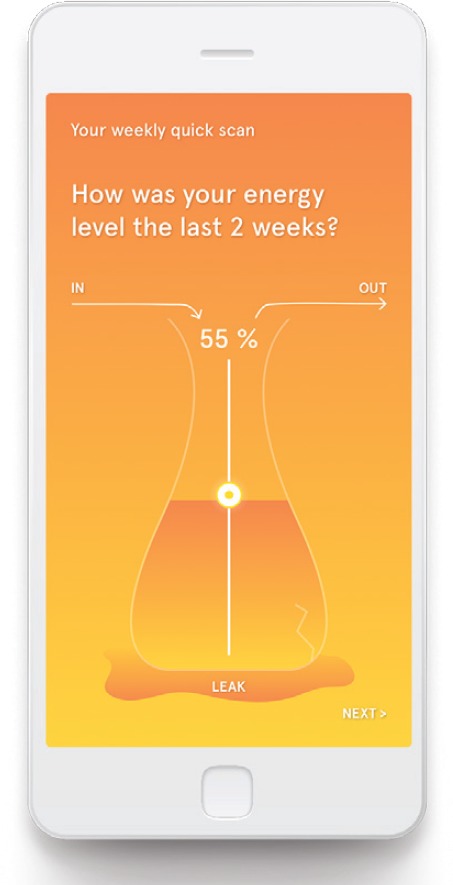
Vase of Energy.

**Figure 2.**
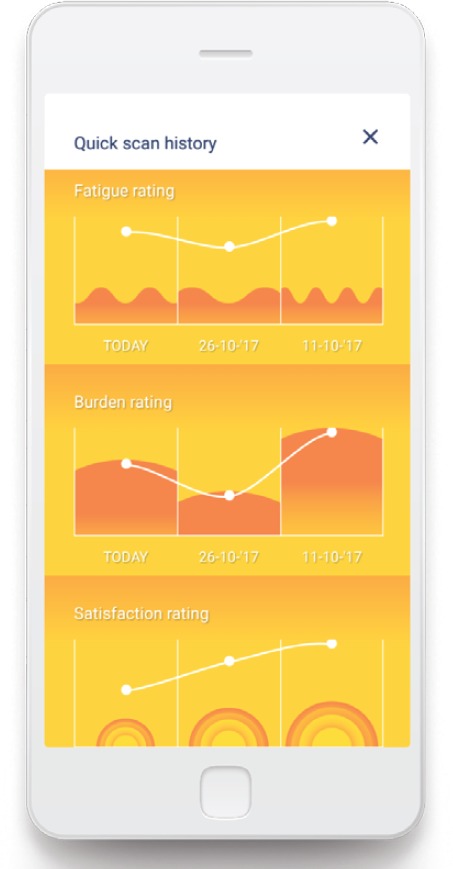
Overview quick scans.

**Figure 3.**
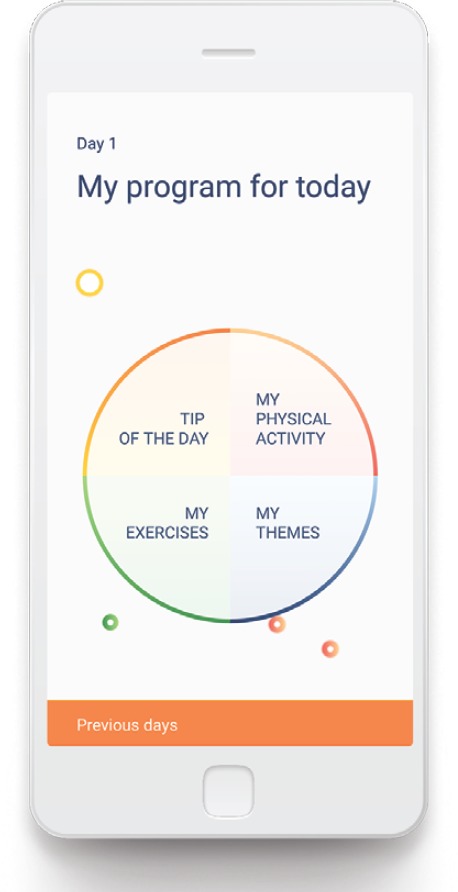
Screenshot of the home page of Untire, showing the Daily Program.

The ‘Untire’ app starts for all users with basic information about CRF and the way the app can help them to monitor their energy and to get more energized. This gives the users the much needed context to start and the motivation to continue with the program. The ‘basics’ finishes by introducing the ‘quick scan’, for which the user is asked every week to provide a VAS rating of their fatigue, the burden of fatigue, satisfaction and happiness. Every two weeks the users will also fill in their own Vase of Energy (see Figure 1). The data from the quick scans are visualized to gain insight in the course of these topics over time (see Figure 2). After the basics the user gets access to the Daily Program, the backbone of this all-embracing app. The Daily Program consists of four components: (1) My physical activity, (2) My themes, (3) My exercises and (4) Tip of the day (see Figure 3). All four components can be accessed from the home page, and will be explained in more detail later.

As stated, the home page prominently resembles the four components of the Daily Program. Each and every day, users can chose for themselves in which order they go through the Daily Program. For example ‘My physical activity’ can be the first daily component of the app. It motivates users to become sufficiently physically active. In the first place users learn to spread their energy better throughout the day and the week. Thereafter they get simple and concrete guidelines to gain more physical strength and condition (f.e. take the stairs instead of the elevator). The aim is to be daily active in a moderate way during at least 30 minutes. In addition, it encourages the user to create a better understanding of how activities gain or cost energy, and to spend energy more consciously. Secondly, ‘My themes’ is the gateway to an extensive program that incorporates the recommendations from NCCN seamlessly: fatigue, boundaries, worry, anxiety, physical activity, sleep, self-care and nutrition. Each theme contains psycho-education (orally and in writing), animations, tips and considerations. Users can pick the theme they are interested in and can interchange easily. They are free to do it at their own pace and in the order that suits them best. Some in-depth texts are placed in the library, for those who are interested in reading more about a certain theme. The third component, ‘My exercises’, contains a variety of stress-reduction exercises (e.g. breathing, body scan, meditation, mindfulness, yoga exercises) and activities that will help the users to identify, manage and release stress every day. Finally, in the ‘Tip of the day’, a focus point, incentive thought, or advice is presented each day. The tips are meant to initiate a good mood and stem especially from positive psychology. On the home page, components that have been accomplished in the Daily Program are highlighted, to motivate the user to engage in the app in all four manners.

Besides the Daily Program, the app provides the means to incorporate a social environment for the user in three ways: (1) a feature to share results of the quick scan with caregivers, (2) the possibility to involve one or more ‘buddies’ (for example partner, friends, colleagues, etc.), and (3) a social platform to allow users to share their experiences with each other.

At this moment ’Untire’ is the first and only app worldwide that offers a concrete, comprehensive and easy-to-use self-management program to cope with cancer-related fatigue.

## Future

An English language version of the app was launched in March 2018. Additionally, it will be translated into several languages so that the app can be distributed throughout the European Union and the USA during 2018. The application can be very interesting and helpful for all caregivers working with (former) cancer patients (medical doctors, nurses, general practitioners, physical therapists, psychologists, etc.) in the context of blended care. Users can carry out this easy-to-use self-management program, while caregivers can eventually give some support through a simple manual that comes with the app.

The app will be evaluated on its effects, use, and mediation. In total 6,000 participants will be involved in a randomized controlled trial (3,000 users and 3,000 participants in the control group). The study will be conducted in several countries in 2018 and 2019 by an independent research group at the University of Groningen. Provisionally, data gathering consists of five online assessments: baseline and at 4, 8, 12, and 24 weeks. The primary outcome will be fatigue reduction throughout the first 12 weeks after baseline. Mediation of associated constructs such as expectations, motivation, mindfulness and sleep difficulties will be studied. Additionally, usage will be studied by means of log data.

## Author note

AJ Kuiper and D Vonk are founders of the Tired of Cancer BV, a social enterprise, by whom the application has been developed. Both have already worked for a long time in the field of psycho-oncology and are very committed to providing fatigued (former) cancer patients with the best help they can get. They want the app to be examined by an independent and prominent scientific research group in order to prevent any conflicts of interest.
